# Supplementary material for: Humans need auditory experience to produce typical volitional nonverbal vocalizations
Source: Commun Psychol. 2024 Jul 18;2:65. doi: 10.1038/s44271-024-00104-6 (PMC11332021; doi:10.1038/s44271-024-00104-6)
Supplement: Supplementary file 2 — Supplementary Information [file 44271_2024_104_MOESM2_ESM.pdf]

Supplementary Information for

Humans need auditory experience to produce typical volitional nonverbal vocalizations

Katarzyna Pisanski*, David Reby & Anna Oleszkiewicz

*Corresponding author. Email: katarzyna.pisanski@cnrs.fr

This PDF file includes:

Supplementary Methods
Supplementary Figures 1 to 2
Supplementary Tables 1 to 20

Supplementary Methods

Instructions were given to all 120 vocalizers in written form prior to voice recording. For deaf participants, instructions were also provided in sign language by a Professional Sign Language Interpreter in a pre-recorded video. The voice recording task was described as follows:

“In a moment, the research assistant will escort you to the recording room where we will record your voice. You will be asked to produce vocal sounds that accompany three different situations. The researcher will present a description of the situation. You will imagine yourself in that scenario and respond to it vocally without using words. We want you to behave completely freely and naturally, so after we provide you with instructions, the assistant will leave the room for a moment.”

The three emotional contexts based on previous work were given as follows:

Aggression: “Imagine that you are walking through a forest with bears. At some point, a mother bear with her cubs emerges on the road ahead of you and begins to run aggressively towards you. Your life is at risk. Produce a vocalization (without using words) to aggressively scare off the bear.”

Fear: “Imagine that you found yourself in a dangerous part of the city after dark. As you pass a dark alley, a masked black-clad person jumps out at you unexpectedly, evoking terror in you. Produce a vocalization (without using words) to express your fear.”

Pain: “Imagine you are experiencing the most intense pain you can think of. Examples are childbirth or a life-threatening injury. Produce a vocalization (without using words) to express your pain.”

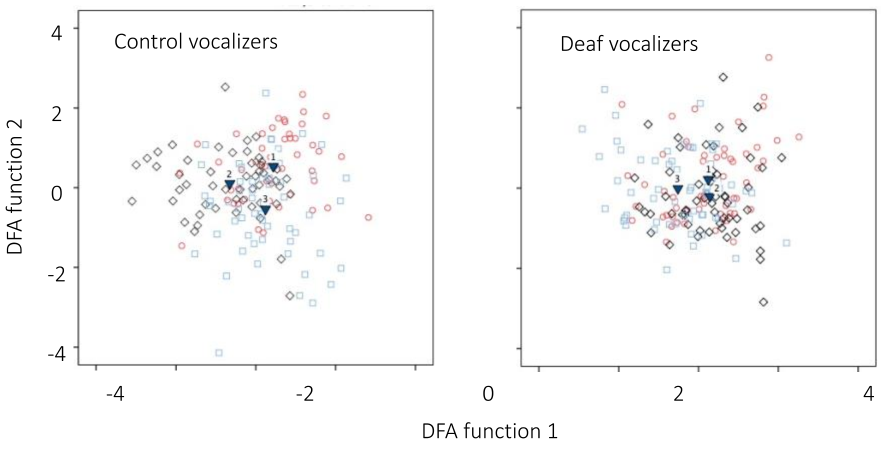

Supplementary Figure 1. Discriminant function analyses (DFA) based on acoustic components derived from principal component analysis (PCA) of all 15 acoustic parameters. The DFA shows a higher degree of acoustic distinctiveness among vocalization types (aggression, pain, fear) produced by typically hearing controls (left) than those produced by deaf vocalizers (right), for whom there is a relatively greater degree of overlap among call types in acoustic space.

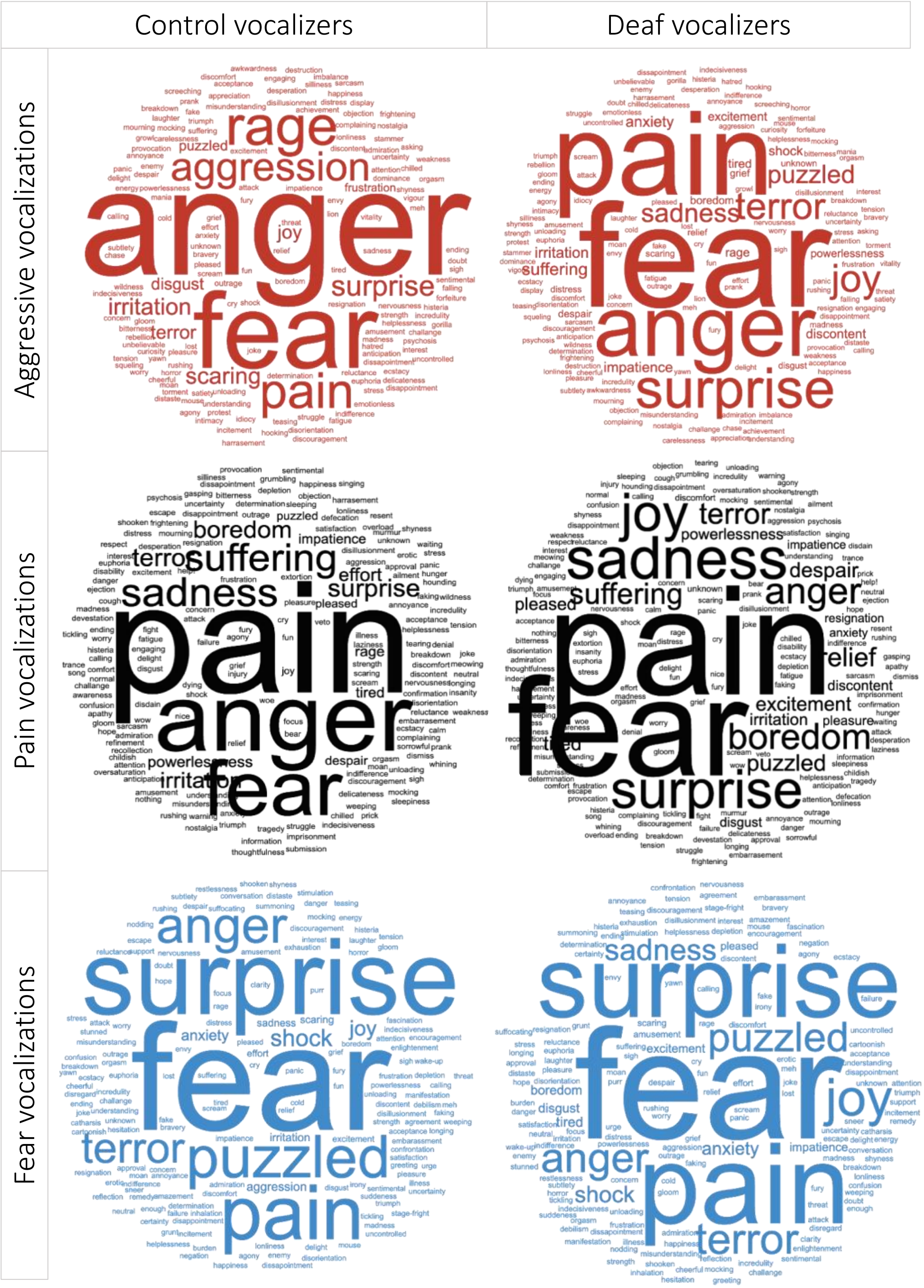

Supplementary Figure 2. Word clouds summarizing one-word labels attributed by listeners to affective vocalizations produced by typically hearing (control, left) and deaf (right) vocalizers, for each emotional context (aggression in red, pain in black, and fear in blue). Data derive from the open-ended emotion identification task (Experiment 2) based on a total of 6120 single word responses collected from 51 participants each labelling 120 vocalizations. The size of each word corresponds to the frequency of its use, with larger words indicating more frequent usage. Word clouds were created in wordcloud.com. Settings: Repetition=off, Zoom=off, Canvas padding=5%, Word margin=4. See also Fig 2d in the main text for number of responses for the top 15 most popular words, and Table S6 for a full list of words.

Supplementary Tables

Supplementary Table 1. Acoustic parameters (*M* mean, *sd* standard deviation) measured from the nonverbal vocalizations of deaf and normally hearing male vocalizers in each emotional context (n = 30 deaf men and 30 control men).

Male vocalizers												
Voice parameter	Aggression				Pain				Fear			
	Control		Deaf		Control		Deaf		Control		Deaf	
	M	*sd*	*M*	*sd*	*M*	*sd*	*M*	*sd*	*M*	*sd*	*M*	*sd*
Mean f_o (Hz)	349.22	97.12	451.42	196.12	369.61	208.11	320.99	146.19	323.45	100.91	446.05	238.06
Min f_o (Hz)	282.03	91.83	354.18	161.54	298.40	173.68	264.54	133.31	247.01	63.68	346.31	169.55
Max f_o (Hz)	381.62	109.37	509.47	225.89	426.02	235.20	379.66	164.55	377.78	163.45	501.05	264.68
f_o CV (Hz)	0.07	0.05	0.10	0.08	0.12	0.15	0.11	0.09	0.11	0.09	0.10	0.09
Inflex 25	3.74	3.50	5.28	2.33	5.28	3.95	4.69	2.83	3.24	2.12	4.24	2.19
Inflex 2	0.39	0.28	0.51	0.37	0.42	0.35	0.66	0.53	0.55	0.40	0.45	0.42
Mean AMP	64.81	8.96	61.72	9.75	58.04	11.61	52.18	11.79	56.20	9.94	58.02	9.39
Max AMP	74.03	8.20	71.83	9.80	67.12	11.25	61.84	12.07	66.80	9.52	68.40	9.48
Int CV	26.70	4.14	27.18	5.67	28.61	6.72	31.13	9.81	30.71	6.45	28.99	4.69
HNR	4.99	3.23	11.05	5.91	7.37	4.33	10.33	5.15	7.04	3.62	10.84	5.99
Jitter	0.03	0.02	0.02	0.01	0.02	0.02	0.02	0.01	0.03	0.02	0.02	0.01
Shimmer	0.16	0.05	0.14	0.06	0.14	0.03	0.13	0.04	0.16	0.05	0.14	0.06
Dur (s)	1.32	0.85	1.73	1.32	2.00	1.74	2.41	2.74	1.27	1.68	1.45	1.24
Dur Vocal (s)	1.14	0.68	1.23	0.72	1.76	1.53	1.99	2.10	1.01	0.99	1.08	0.73
NLP (%)	45.31	30.34	7.52	13.70	36.04	25.91	4.86	11.41	6.20	15.12	5.25	13.53

Supplementary Table 2. Acoustic parameters (*M* mean, *sd* standard deviation) measured from the vocalizations of deaf and normally hearing female vocalizers in each emotional context (n = 30 deaf women and 30 control women).

Female vocalizers

Voice parameter	Aggression				Pain				Fear			
	Control		Deaf		Control		Deaf		Control		Deaf	
	M	*sd*	*M*	*sd*	*M*	*sd*	*M*	*sd*	*M*	*sd*	*M*	*sd*
Mean f_o (Hz)	512.49	291.88	591.74	306.46	525.74	162.26	565.86	294.58	794.29	298.17	729.06	402.65
Min f_o (Hz)	394.57	231.37	438.79	227.80	407.74	122.34	413.09	185.40	637.13	265.42	540.59	285.60
Max f_o (Hz)	589.45	369.86	697.74	346.98	603.52	219.67	666.73	368.33	886.92	328.55	837.98	463.83
f_o CV (Hz)	0.09	0.07	0.11	0.06	0.09	0.08	0.11	0.08	0.08	0.06	0.11	0.09
Inflex 25	5.67	3.85	5.11	2.56	6.03	3.16	5.62	2.86	5.25	2.77	4.64	2.58
Inflex 2	0.75	0.41	0.71	0.46	0.78	0.45	0.63	0.46	0.57	0.50	0.57	0.43
Mean AMP	58.68	9.68	56.34	8.81	54.89	10.72	52.86	12.23	56.62	11.82	56.58	11.59
Max AMP	67.05	9.65	65.62	8.67	63.30	10.36	62.14	12.43	65.26	11.25	66.68	11.25
Int CV	28.51	5.40	28.28	5.56	29.14	7.85	30.16	8.28	31.31	7.89	27.80	7.84
HNR	7.84	5.58	11.41	5.98	13.57	5.88	13.53	5.31	10.83	5.98	11.93	4.52
Jitter	0.03	0.02	0.02	0.01	0.01	0.01	0.01	0.01	0.02	0.02	0.02	0.02
Shimmer	0.14	0.04	0.12	0.06	0.11	0.05	0.11	0.05	0.11	0.04	0.12	0.04
Dur (s)	1.72	0.98	2.24	2.00	2.42	1.87	2.64	2.71	1.19	0.72	2.30	2.40
Dur Vocal (s)	1.61	0.90	1.74	1.38	2.26	1.74	2.01	2.16	1.11	0.68	1.44	1.04
NLP (%)	40.46	32.93	9.00	16.37	15.04	21.03	3.32	11.50	10.84	21.69	3.71	10.04

Supplementary Table 3. Sample descriptives of adult listeners taking part in four independent perception experiments.

		N listeners	Female listeners		Male listeners	
Experiment:			Mean age	Age range	Mean age	Age range
1	Forced-choice emotion classification	139 (82 female)	28.3	16 - 55	30.4	18 - 55
2	Open-ended emotion identification	51 (36 female)	27.6	18 - 52	26.1	19 - 49
3	Authenticity identification	117 (61 female)	30.1	19 - 57	32.9	18 - 59
4	Deafness detection	137 (85 female)	30.3	17 - 60	29.3	16 - 55
Total		444 (264 female)	29.1	16-60	29.7	16-59

Supplementary Table 4. Principal Component Analysis (PCA) of 15 vocal parameters.

Voice parameter	Principal Component [eigenvalue, variance explained]				
	1	2	3	4	5
	[4.6, 30.3%]	[2.8, 18.5%]	[2.3, 15%]	[1.8, 12.2%]	[1.1, 7.4%]
Mean f_o	**.967**	.183	-.093	.018	.046
Min f_o	**.896**	.205	-.165	-.146	.115
Max f_o	**.970**	.153	-.042	.136	.019
f_o CV	.195	-.158	.307	.598	-.265
Inflex 25	.113	.060	-.040	.200	**.834**
Inflex 2	.047	-.024	-.189	.103	**.796**
Mean AMP	.245	**.942**	-.057	-.113	.010
Max AMP	.253	**.938**	-.053	-.076	-.058
Int CV	-.119	**-.835**	.167	-.418	-.136
HNR	.261	.090	**-.819**	.065	.299
Jitter	.137	-.169	**.889**	-.016	-.016
Shimmer	-.172	-.074	**.813**	-.074	-.190
Dur	-.066	.068	-.154	**.877**	.268
Dur Vocal	-.072	.114	-.128	**.829**	.425
% NLP	-.177	.398	.552	.085	.276

Factor loading scores exceeding ±0.71 are bolded, indicating that at least 50% or more of the variance in the given voice parameter is accounted for in the given principal component. Acronyms: f_o = fundamental frequency; CV = coefficient of variation; inflex 25 = minor f_o inflections; inflex 2 = major f_o inflections; AMP = amplitude; Int = intensity; HNR = harmonics-to-noise ratio; Dur = duration; NLP = nonlinear acoustic phenomena.

Supplementary Table 5. Discriminant Function Analysis (DFA) of acoustic principal components examining correct classification of vocalizations based on their intended emotion, for deaf and control vocalizers.

		Model predicted emotion			
	Intended emotion	Aggression	Fear	Pain	Overall Correct
Control vocalizers	Aggression	**57.5**	25.5	17.0	50.3
	Fear	34.0	**47.2**	18.9	
	Pain	26.3	26.3	**47.4**	
Deaf vocalizers	Aggression	**36.8**	31.6	31.6	42.7
	Fear	26.8	**42.9**	30.4	
	Pain	19.0	32.8	**48.3**	

Cross-validated correct classification (%) of intended emotion as a function of the underlying acoustic parameters (principal components) of vocalizations (see Table S2), shown separately for normally hearing controls and deaf vocalizers. Bolded values indicate the percentage of correct classification for each intended emotion.

Supplementary Table 6. One-word responses in the open-ended emotion identification task (Experiment 2) for vocalizations produced by deaf vocalizers and normally hearing controls, and the coded valence of each word (positive, neutral/ambiguous, negative).

**Aggression**			
	Frequency of use when describing:		Valence
One-word response (translated)	Deaf vocalizers	Controls	
acceptance	1	0	Positive
achievement	0	1	Positive
admiration	1	1	Positive
amusement	7	5	Positive
appreciation	0	1	Positive
bravery	0	5	Positive
cheerful	1	0	Positive
chilled	1	0	Positive
delight	3	0	Positive
determination	1	5	Positive
ecstacy	1	1	Positive
engaging	1	0	Positive
euphoria	2	1	Positive
excitement	12	7	Positive
fun	4	2	Positive
happiness	1	0	Positive
joke	0	1	Positive
joy	38	23	Positive
laughter	3	0	Positive
nostalgia	1	0	Positive
orgasm	1	0	Positive
pleased	6	5	Positive
pleasure	1	3	Positive
prank	2	0	Positive
relief	9	6	Positive
satiety	0	1	Positive
sentimental	1	0	Positive
silliness	1	0	Positive
triumph	0	2	Positive
understanding	0	1	Positive
vigour	0	1	Positive
vitality	1	2	Positive
anticipation	2	2	Neutral or Ambiguous
asking	1	0	Neutral or Ambiguous
attention	2	2	Neutral or Ambiguous
calling	0	1	Neutral or Ambiguous
challenge	1	1	Neutral or Ambiguous
chase	1	0	Neutral or Ambiguous
cold	0	1	Neutral or Ambiguous

curiosity	3	1	Neutral or Ambiguous
delicateness	2	0	Neutral or Ambiguous
disorientation	4	0	Neutral or Ambiguous
display	1	0	Neutral or Ambiguous
dominance	0	2	Neutral or Ambiguous
doubt	1	0	Neutral or Ambiguous
effort	5	7	Neutral or Ambiguous
emotionless	1	0	Neutral or Ambiguous
ending	1	0	Neutral or Ambiguous
energy	1	1	Neutral or Ambiguous
falling	1	0	Neutral or Ambiguous
forfeiture	1	0	Neutral or Ambiguous
gorilla	1	0	Neutral or Ambiguous
growl	1	0	Neutral or Ambiguous
hooking	1	0	Neutral or Ambiguous
incitement	1	0	Neutral or Ambiguous
indecisiveness	0	1	Neutral or Ambiguous
indifference	1	0	Neutral or Ambiguous
interest	1	1	Neutral or Ambiguous
intimacy	1	0	Neutral or Ambiguous
lion	0	1	Neutral or Ambiguous
mania	0	1	Neutral or Ambiguous
meh	1	0	Neutral or Ambiguous
moan	3	0	Neutral or Ambiguous
mouse	1	0	Neutral or Ambiguous
objection	1	1	Neutral or Ambiguous
puzzled	25	17	Neutral or Ambiguous
rebellion	0	1	Neutral or Ambiguous
rushing	2	2	Neutral or Ambiguous
scream	2	1	Neutral or Ambiguous
screeching	1	0	Neutral or Ambiguous
shock	13	8	Neutral or Ambiguous
shyness	0	1	Neutral or Ambiguous
sigh	1	0	Neutral or Ambiguous
squealing	1	0	Neutral or Ambiguous
strength	0	3	Neutral or Ambiguous
subtlety	0	1	Neutral or Ambiguous
surprise	55	35	Neutral or Ambiguous
unbelievable	0	1	Neutral or Ambiguous
uncertainty	5	1	Neutral or Ambiguous
unknown	9	4	Neutral or Ambiguous
unloading	2	2	Neutral or Ambiguous
wildness	1	2	Neutral or Ambiguous
yawn	0	1	Neutral or Ambiguous
aggression	7	49	Negative
agony	1	0	Negative
anger	91	293	Negative
annoyance	2	1	Negative
anxiety	15	6	Negative

attack	1	3	Negative
awkwardness	1	0	Negative
bitterness	1	1	Negative
boredom	11	9	Negative
breakdown	2	0	Negative
carelessness	0	1	Negative
complaining	0	1	Negative
concern	3	0	Negative
cry	3	2	Negative
despair	10	2	Negative
desperation	1	1	Negative
destruction	1	0	Negative
disappointment	4	0	Negative
discomfort	7	0	Negative
discontent	13	7	Negative
discouragement	2	0	Negative
disgust	9	20	Negative
disillusionment	7	1	Negative
disappointment	0	1	Negative
distaste	1	0	Negative
distress	9	1	Negative
enemy	1	2	Negative
envy	0	2	Negative
fake	0	1	Negative
fatigue	4	0	Negative
fear	193	128	Negative
frightening	1	0	Negative
frustration	5	14	Negative
fury	1	8	Negative
gloom	1	2	Negative
grief	10	2	Negative
harassment	1	0	Negative
hatred	1	3	Negative
helplessness	5	2	Negative
hysteria	1	1	Negative
horror	0	1	Negative
idiocy	2	1	Negative
imbalance	1	0	Negative
impatience	14	7	Negative
incredulity	2	1	Negative
irritation	15	28	Negative
loneliness	0	3	Negative
lost	1	1	Negative
madness	8	3	Negative
misunderstanding	1	1	Negative
mocking	1	1	Negative
mourning	1	0	Negative
nervousness	2	6	Negative
outrage	4	10	Negative

pain	133	60	Negative
panic	1	1	Negative
powerlessness	11	5	Negative
protest	0	1	Negative
provocation	3	1	Negative
psychosis	0	1	Negative
rage	12	67	Negative
reluctance	7	2	Negative
resignation	7	7	Negative
sadness	26	5	Negative
sarcasm	1	0	Negative
scaring	9	28	Negative
stammer	0	1	Negative
stress	2	1	Negative
struggle	0	1	Negative
suffering	17	4	Negative
teasing	0	1	Negative
tension	1	0	Negative
terror	37	23	Negative
threat	0	4	Negative
tired	12	2	Negative
torment	1	1	Negative
uncontrolled	1	0	Negative
weakness	2	0	Negative
worry	1	0	Negative

Fear

	Frequency of use when describing:		Valence
One-word response (translated)	Deaf vocalizers	Controls	
acceptance	1	1	Positive
admiration	5	4	Positive
agreement	0	1	Positive
amusement	9	3	Positive
approval	0	1	Positive
bravery	0	3	Positive
cheerful	1	0	Positive
clarity	0	3	Positive
delight	2	1	Positive
determination	0	2	Positive
ecstasy	0	1	Positive
encouragement	1	0	Positive
enlightenment	0	1	Positive
euphoria	4	2	Positive
excitement	12	10	Positive
fascination	0	1	Positive
fun	2	1	Positive
happiness	2	0	Positive
hope	0	1	Positive
joke	2	0	Positive

joy	52	24	Positive
laughter	2	1	Positive
orgasm	1	2	Positive
pleased	11	8	Positive
pleasure	5	1	Positive
relief	5	5	Positive
remedy	0	1	Positive
satisfaction	1	1	Positive
sentimental	0	1	Positive
support	0	1	Positive
tickling	0	1	Positive
triumph	0	1	Positive
understanding	1	1	Positive
amazement	1	2	Neutral or Ambiguous
attention	0	2	Neutral or Ambiguous
calling	1	1	Neutral or Ambiguous
cartoonish	1	0	Neutral or Ambiguous
catharsis	1	0	Neutral or Ambiguous
certainty	1	0	Neutral or Ambiguous
challenge	0	2	Neutral or Ambiguous
cold	0	1	Neutral or Ambiguous
conversation	1	0	Neutral or Ambiguous
disorientation	3	0	Neutral or Ambiguous
doubt	1	0	Neutral or Ambiguous
effort	9	11	Neutral or Ambiguous
ending	1	0	Neutral or Ambiguous
energy	1	0	Neutral or Ambiguous
enough	0	1	Neutral or Ambiguous
erotic	1	0	Neutral or Ambiguous
escape	2	0	Neutral or Ambiguous
faking	2	0	Neutral or Ambiguous
focus	0	2	Neutral or Ambiguous
greeting	0	1	Neutral or Ambiguous
grunt	1	0	Neutral or Ambiguous
hesitation	0	2	Neutral or Ambiguous
incitement	0	1	Neutral or Ambiguous
indecisiveness	1	1	Neutral or Ambiguous
indifference	2	1	Neutral or Ambiguous
inhalation	0	2	Neutral or Ambiguous
interest	2	1	Neutral or Ambiguous
irony	1	0	Neutral or Ambiguous
manifestation	0	1	Neutral or Ambiguous
meh	1	0	Neutral or Ambiguous
moan	2	0	Neutral or Ambiguous
mouse	1	0	Neutral or Ambiguous
negation	1	0	Neutral or Ambiguous
neutral	1	0	Neutral or Ambiguous
nodding	1	0	Neutral or Ambiguous
purr	0	1	Neutral or Ambiguous

puzzled	41	67	Neutral or Ambiguous
reflection	1	0	Neutral or Ambiguous
rushing	3	0	Neutral or Ambiguous
scream	4	1	Neutral or Ambiguous
shock	23	27	Neutral or Ambiguous
shyness	1	0	Neutral or Ambiguous
sigh	2	2	Neutral or Ambiguous
stimulation	2	0	Neutral or Ambiguous
strength	0	3	Neutral or Ambiguous
stunned	0	1	Neutral or Ambiguous
subtlety	1	0	Neutral or Ambiguous
suddenness	0	1	Neutral or Ambiguous
summoning	0	1	Neutral or Ambiguous
surprise	102	162	Neutral or Ambiguous
uncertainty	7	0	Neutral or Ambiguous
unknown	2	2	Neutral or Ambiguous
unloading	3	6	Neutral or Ambiguous
urge	1	0	Neutral or Ambiguous
wake-up	0	1	Neutral or Ambiguous
yawn	1	0	Neutral or Ambiguous
aggression	8	14	Negative
agony	2	0	Negative
anger	42	58	Negative
annoyance	0	2	Negative
anxiety	18	17	Negative
attack	1	1	Negative
boredom	13	5	Negative
breakdown	1	0	Negative
burden	1	0	Negative
concern	3	2	Negative
confrontation	0	1	Negative
confusion	1	0	Negative
cry	1	1	Negative
danger	1	0	Negative
debilism	0	1	Negative
depletion	3	1	Negative
despair	8	0	Negative
disappointment	1	1	Negative
discomfort	8	4	Negative
discontent	7	3	Negative
discouragement	1	1	Negative
disgust	13	3	Negative
disillusionment	2	2	Negative
disregard	1	0	Negative
disappointment	1	0	Negative
distaste	1	0	Negative
distress	7	5	Negative
embarrassment	0	1	Negative
enemy	0	1	Negative

envy	0	1	Negative
exhaustion	1	2	Negative
failure	0	1	Negative
fake	0	1	Negative
fear	226	266	Negative
frustration	3	4	Negative
fury	0	1	Negative
gloom	1	0	Negative
grief	2	2	Negative
helplessness	3	0	Negative
hysteria	1	0	Negative
horror	0	1	Negative
illness	1	0	Negative
impatience	12	9	Negative
incredulity	1	2	Negative
irritation	5	13	Negative
longing	1	0	Negative
loneliness	1	1	Negative
lost	1	0	Negative
madness	6	0	Negative
misunderstanding	1	1	Negative
mocking	1	1	Negative
nervousness	0	1	Negative
outrage	5	2	Negative
pain	112	86	Negative
panic	8	8	Negative
powerlessness	6	5	Negative
rage	3	5	Negative
reluctance	3	0	Negative
resignation	2	0	Negative
restlessness	1	0	Negative
sadness	30	12	Negative
scaring	9	12	Negative
shaken	1	0	Negative
sneer	1	0	Negative
stage-fright	1	0	Negative
stress	1	0	Negative
suffering	7	8	Negative
suffocating	0	1	Negative
teasing	1	0	Negative
tension	1	0	Negative
terror	43	49	Negative
threat	1	0	Negative
tired	14	8	Negative
uncontrolled	1	0	Negative
weeping	1	0	Negative
worry	3	2	Negative
**Pain**			
	Frequency of use when describing:		Valence

One-word response (translated)	Deaf vocalizers	Controls	
acceptance	1	2	Positive
admiration	2	2	Positive
amusement	4	0	Positive
approval	0	1	Positive
calm	5	0	Positive
chilled	1	0	Positive
comfort	0	1	Positive
confirmation	1	3	Positive
delight	6	4	Positive
determination	0	3	Positive
ecstasy	3	3	Positive
engaging	1	2	Positive
euphoria	2	0	Positive
excitement	18	5	Positive
fun	1	0	Positive
happiness	2	0	Positive
hope	1	0	Positive
joke	1	0	Positive
joy	48	10	Positive
nice	1	0	Positive
nostalgia	1	0	Positive
orgasm	2	3	Positive
pleased	16	12	Positive
pleasure	12	3	Positive
prank	1	0	Positive
refinement	0	1	Positive
relief	24	1	Positive
respect	1	0	Positive
satisfaction	3	5	Positive
sentimental	1	0	Positive
silliness	1	0	Positive
singing	1	0	Positive
tickling	1	0	Positive
triumph	0	1	Positive
understanding	7	4	Positive
anticipation	1	0	Neutral or Ambiguous
attention	1	0	Neutral or Ambiguous
awareness	1	0	Neutral or Ambiguous
bear	1	0	Neutral or Ambiguous
calling	1	1	Neutral or Ambiguous
challenge	1	0	Neutral or Ambiguous
cough	1	0	Neutral or Ambiguous
delicateness	1	1	Neutral or Ambiguous
denial	1	0	Neutral or Ambiguous
disability	2	0	Neutral or Ambiguous
disorientation	1	1	Neutral or Ambiguous
effort	1	19	Neutral or Ambiguous
ejection	1	0	Neutral or Ambiguous

ending	0	1	Neutral or Ambiguous
erotic	1	0	Neutral or Ambiguous
escape	1	0	Neutral or Ambiguous
faking	2	0	Neutral or Ambiguous
fight	0	1	Neutral or Ambiguous
focus	0	1	Neutral or Ambiguous
gasping	1	0	Neutral or Ambiguous
hunger	1	0	Neutral or Ambiguous
indecisiveness	2	0	Neutral or Ambiguous
indifference	3	3	Neutral or Ambiguous
information	1	0	Neutral or Ambiguous
interest	1	0	Neutral or Ambiguous
meowing	1	0	Neutral or Ambiguous
moan	2	1	Neutral or Ambiguous
murmur	1	0	Neutral or Ambiguous
neutral	1	0	Neutral or Ambiguous
normal	0	1	Neutral or Ambiguous
nothing	2	0	Neutral or Ambiguous
objection	0	1	Neutral or Ambiguous
puzzled	21	12	Neutral or Ambiguous
recollection	0	1	Neutral or Ambiguous
rushing	1	0	Neutral or Ambiguous
scream	2	3	Neutral or Ambiguous
shock	2	4	Neutral or Ambiguous
shyness	1	0	Neutral or Ambiguous
sigh	1	0	Neutral or Ambiguous
sleepiness	1	0	Neutral or Ambiguous
sleeping	0	1	Neutral or Ambiguous
song	1	0	Neutral or Ambiguous
strength	0	3	Neutral or Ambiguous
surprise	49	26	Neutral or Ambiguous
tearing	0	2	Neutral or Ambiguous
thoughtfulness	2	0	Neutral or Ambiguous
trance	1	0	Neutral or Ambiguous
uncertainty	3	1	Neutral or Ambiguous
unknown	8	1	Neutral or Ambiguous
unloading	0	1	Neutral or Ambiguous
veto	0	1	Neutral or Ambiguous
waiting	1	0	Neutral or Ambiguous
warning	0	1	Neutral or Ambiguous
wildness	1	0	Neutral or Ambiguous
wow	1	0	Neutral or Ambiguous
aggression	4	7	Negative
agony	0	4	Negative
ailment	0	1	Negative
anger	34	125	Negative
annoyance	1	4	Negative
anxiety	11	1	Negative
apathy	1	0	Negative

attack	0	1	Negative
bitterness	1	1	Negative
boredom	33	25	Negative
breakdown	1	4	Negative
childish	1	0	Negative
complaining	4	1	Negative
concern	4	2	Negative
confusion	1	0	Negative
cry	3	0	Negative
danger	1	0	Negative
defecation	0	2	Negative
depletion	2	1	Negative
despair	19	13	Negative
desperation	0	2	Negative
devastation	1	0	Negative
disappointment	2	3	Negative
discomfort	8	4	Negative
discontent	13	4	Negative
discouragement	4	1	Negative
disdain	0	1	Negative
disgust	12	6	Negative
disillusionment	7	4	Negative
dismiss	0	1	Negative
disappointment	2	0	Negative
distress	4	0	Negative
dying	0	1	Negative
embarrassment	0	2	Negative
extortion	1	1	Negative
failure	1	2	Negative
fatigue	3	3	Negative
fear	126	100	Negative
frightening	0	1	Negative
frustration	3	5	Negative
fury	0	3	Negative
gloom	2	0	Negative
grief	5	5	Negative
grumbling	1	0	Negative
harassment	1	0	Negative
help!	0	1	Negative
helplessness	6	2	Negative
hysteria	1	1	Negative
hounding	1	0	Negative
illness	1	2	Negative
impatience	11	14	Negative
imprisonment	1	0	Negative
incredulity	1	0	Negative
injury	0	1	Negative
insanity	2	0	Negative
irritation	15	23	Negative

laziness	0	1	Negative
longing	1	0	Negative
loneliness	0	1	Negative
madness	3	0	Negative
misunderstanding	2	1	Negative
mocking	2	0	Negative
mourning	0	2	Negative
nervousness	2	3	Negative
outrage	1	3	Negative
overload	0	1	Negative
oversaturation	1	0	Negative
pain	166	295	Negative
panic	4	0	Negative
powerlessness	14	16	Negative
prick	1	0	Negative
provocation	1	0	Negative
psychosis	1	0	Negative
rage	3	15	Negative
reluctance	5	2	Negative
resent	1	0	Negative
resignation	11	8	Negative
sadness	54	42	Negative
sarcasm	1	1	Negative
scaring	4	3	Negative
shaken	1	0	Negative
sorrowful	0	1	Negative
stress	2	0	Negative
struggle	1	1	Negative
submission	1	0	Negative
suffering	27	40	Negative
tension	1	0	Negative
terror	27	26	Negative
tired	18	15	Negative
tragedy	0	1	Negative
weakness	1	0	Negative
weeping	2	1	Negative
whining	1	0	Negative
woe	0	1	Negative
worry	5	1	Negative

Note: Two researchers independently and blindly coded the valence of each response as negative, neutral / ambiguous, or positive. Responses coded as “neutral or ambiguous” are those that either do not carry a strong positive or negative valance, or those whose valance cannot be determined because the word is affectively ambiguous (e.g., can have multiple meanings).

Supplementary Table 7. Linear Mixed Models: Acoustic differences between deaf and normally hearing (control) vocalizers in aggressive vocalizations.

Acoustic parameter	Male vocalizers			Female vocalizers		
**Linear Mixed Models**	*df1, df2*	*F*	*P*	*df1, df2*	*F*	*P*
Mean f_o	1, 51	5.4	.024 *	1, 50	0.9	.349
Min f_o	1, 51	3.8	.058 †	1, 50	0.5	.493
Max f_o	1, 51	6.4	.014 *	1, 50	1.2	.283
f_o CV	1, 58	3.0	.089 †	1, 58	1.0	.320
Inflex 25	1, 51	3.7	.061 †	1, 50	0.4	.538
Inflex 2	1, 51	1.7	.198	1, 50	0.1	.736
Mean AMP	1, 58	1.6	.207	1, 58	1.0	.331
Max AMP	1, 58	0.9	.349	1, 58	0.4	.548
Int CV	1, 58	0.1	.706	1, 58	0.1	.873
HNR	1, 58	24.3	<.001 ***	1, 58	5.7	.020 *
Jitter	1, 58	7.8	.007 **	1, 58	4.4	.039 *
Shimmer	1, 54	2.5	.119	1, 56	2.9	.092 †
Dur	1, 58	2.1	.154	1, 58	1.6	.205
Dur Vocal	1, 58	0.2	.650	1, 58	0.2	.672
% NLP	1, 58	38.6	<.001 ***	1, 58	22.0	<.001 ***

	Control		Deaf		Control		Deaf	
**Estimated Marginal Means**	EMM	*sem*	EMM	*sem*	EMM	*sem*	EMM	*sem*
**Mean f_o (Hz)**	**349.2**	**32.5**	**451.4**	**29.6**	512.5	62.6	591.7	55.7
Min f_o (Hz)	282.0	27.5	354.2	25.0	394.6	47.8	438.8	42.6
**Max f_o (Hz)**	**381.6**	**37.3**	**509.5**	**33.9**	589.4	74.5	697.7	66.3
f_o CV (Hz)	0.07	0.01	.102	0.01	0.09	0.01	0.11	0.01
Inflex 25	3.7	0.6	5.3	0.5	5.7	0.7	5.1	0.6
Inflex 2	0.38	0.06	0.51	0.06	0.75	0.09	0.71	0.08
Mean AMP (dB)	64.8	1.7	61.7	1.7	58.7	1.7	56.3	1.7
Max AMP (dB)	74.0	1.6	71.8	1.6	67.0	1.7	65.6	1.7
Int CV (dB)	26.7	0.9	27.2	0.9	28.5	1.0	28.3	1.0
**HNR (dB)**	**5.0**	**0.9**	**11.1**	**0.9**	**7.8**	**1.1**	**11.4**	**1.1**
**Jitter (Hz)**	**0.030**	**0.003**	**0.019**	**0.003**	**0.029**	**0.004**	**0.019**	**0.004**
Shimmer (dB)	0.162	0.011	0.139	0.010	0.141	0.009	0.119	0.009
Dur (s)	1.3	0.2	1.7	0.2	1.7	0.3	2.2	0.3
Dur Vocal (s)	1.1	0.1	1.2	0.1	1.6	0.2	1.7	0.2
**% NLP**	**45.3**	**4.3**	**7.5**	**4.3**	**40.5**	**4.7**	**9.0**	**4.7**

Linear mixed models (LMMs) and resulting estimated marginal means (EMMs ± standard error of the mean, *sem*; significant differences bolded) comparing voice parameters between deaf and normally hearing men and women in the aggression context. All models, dependent variable: the given voice parameter; random variable (with intercept): vocalizer ID; fixed variables: deafness (impaired hearing or normal hearing). For all LMMs, model intercepts were significant at $p<.001$. Significant effects in LMMs were further examined using pairwise tests with Šidák correction for multiple comparisons. All tests two-tailed, alpha 0.05, where *** $p<.001$; ** $p<.01$; * $p<.05$; †$p<.10$. Data derive from $n=120$ vocalizers with an even sex ratio (60 deaf; 60 controls); for a small number of voice stimuli, specific acoustic parameters could not be measured, for instance, f_o parameters in the presence of severe deterministic chaos (see Methods).

Supplementary Table 8. Linear Mixed Models: Acoustic differences between deaf and normally hearing (control) vocalizers in pain vocalizations.

Acoustic parameter	Male vocalizers			Female vocalizers		
	df1, df2	*F*	*P*	*df1, df2*	*F*	*P*
Mean f_o	1, 54	1.0	.316	1, 58	0.4	.516
Min f_o	1, 54	0.7	.417	1, 58	0.1	.895
Max f_o	1, 54	0.7	.397	1, 58	0.7	.423
f_o CV	1, 58	0.1	.807	1, 58	1.3	.252
Inflex 25	1, 54	0.4	.520	1, 58	0.3	.599
Inflex 2	1, 54	4.3	.043 *	1, 58	1.6	.206
Mean AMP	1, 58	3.8	.057 †	1, 58	0.5	.497
Max AMP	1, 58	3.1	.085 †	1, 58	0.2	.696
Int CV	1, 58	1.3	.251	1, 58	0.2	.626
HNR	1, 58	5.8	.019 *	1, 58	0.1	.978
Jitter	1, 58	3.6	.063 †	1, 58	0.2	.671
Shimmer	1, 56	1.0	.328	1, 58	0.3	.605
Dur	1, 58	0.5	.501	1, 58	0.1	.718
Dur Vocal	1, 58	0.2	.633	1, 58	0.2	.623
% NLP	1, 58	36.4	<.001 ***	1, 58	7.2	.010 **

	Control		Deaf		Control		Deaf	
**Estimated Marginal Means**	EMM	*sem*	EMM	*sem*	EMM	*sem*	EMM	*sem*
Mean f_o (Hz)	369.6	34.0	321.0	34.0	525.7	43.4	565.9	43.4
Min f_o (Hz)	298.4	29.3	264.5	29.3	407.7	28.7	413.1	28.7
Max f_o (Hz)	426.0	38.4	379.7	38.4	603.5	55.4	666.7	55.4
f_o CV (Hz)	0.12	0.02	0.11	0.02	0.09	0.01	0.11	0.01
Inflex 25	5.3	0.6	4.7	0.6	6.0	0.6	5.6	0.6
**Inflex 2**	**0.42**	**0.09**	**0.66**	**0.09**	0.78	0.08	0.63	0.08
Mean AMP (dB)	58.0	2.1	52.2	2.1	54.9	2.1	52.9	2.1
Max AMP (dB)	67.1	2.1	61.8	2.1	63.3	2.1	62.1	2.1
Int CV (dB)	28.6	1.5	31.1	1.5	29.1	1.5	30.2	1.5
**HNR (dB)**	**7.4**	**0.9**	**10.3**	**0.9**	13.6	1.0	13.5	1.0
Jitter (Hz)	0.025	0.003	0.017	0.003	0.013	0.002	0.015	0.002
Shimmer (dB)	0.142	0.007	0.133	0.007	0.105	0.009	0.112	0.009
Dur (s)	2.0	0.4	2.4	0.4	2.4	0.4	2.6	0.4
Dur Vocal (s)	1.8	0.3	2.0	0.3	2.3	0.4	2.0	0.4
**% NLP**	**36.0**	**3.7**	**4.9**	**3.7**	**15.0**	**3.1**	**3.3**	**3.1**

Linear mixed models (LMMs) and resulting estimated marginal means (EMMs ± standard error of the mean, *sem;* significant differences bolded) comparing voice parameters between deaf and normally hearing men and women in the pain context. All models, dependent variable: the given voice parameter; random variable (with intercept): vocalizer ID; fixed variables: deafness (impaired hearing or normal hearing). For all LMMs, model intercepts were significant at p<.001. Significant effects in LMMs were further examined using pairwise tests with Šidák correction for multiple comparisons. All tests two-tailed, alpha 0.05, where *** p<.001; ** p<.01; * p<.05; †p<.10. Data derive from n=120 vocalizers with an even sex ratio (60 deaf; 60 controls); for a small number of voice stimuli, specific acoustic parameters could not be measured, for instance, f_o parameters in the presence of severe deterministic chaos (see Methods).

Supplementary Table 9. Linear Mixed Models: Acoustic differences between deaf and normally hearing (control) vocalizers in fear vocalizations.

Acoustic parameter	Male vocalizers			Female vocalizers		
**Linear Mixed Models**	*df1, df2*	*F*	*P*	*df1, df2*	*F*	*P*
Mean f_o	1, 54	6.0	.018 *	1, 54	0.5	.494
Min f_o	1, 54	7.9	.007 **	1, 54	1.7	.196
Max f_o	1, 54	4.2	.044 *	1, 54	0.2	.651
f_o CV	1, 58	0.2	.693	1, 57	2.5	.117
Inflex 25	1, 54	3.0	.089 †	1, 54	0.7	.398
Inflex 2	1, 54	0.8	.375	1, 54	0.1	.986
Mean AMP	1, 58	0.5	.469	1, 58	0.1	.990
Max AMP	1, 58	0.4	.519	1, 58	0.2	.627
Int CV	1, 58	1.4	.242	1, 58	3.0	.089 †
HNR	1, 58	8.9	.004 **	1, 58	0.6	.426
Jitter	1, 58	4.7	.034 *	1, 58	0.1	.712
Shimmer	1, 55	2.5	.122	1, 56	0.9	.340
Dur	1, 58	0.2	.641	1, 58	5.8	.019
Dur Vocal	1, 58	0.1	.787	1, 58	2.1	.155
% NLP	1, 58	0.1	.799	1, 58	2.7	.080

	Control		Deaf		Control		Deaf	
**Estimated Marginal Means**	EMM	*sem*	EMM	*sem*	EMM	*sem*	EMM	*sem*
**Mean f_o (Hz)**	**323.4**	**36.8**	**446.1**	**34.2**	794.3	70.0	729.1	70.0
**Min f_o (Hz)**	**247.0**	**25.8**	**346.3**	**24.0**	637.1	52.0	540.6	52.1
**Max f_o (Hz)**	**377.8**	**43.8**	**501.1**	**40.8**	886.9	76.0	838.0	76.0
f_o CV (Hz)	0.11	0.02	0.10	0.02	0.08	0.01	0.11	0.01
Inflex 25	3.2	0.4	4.2	0.4	5.3	0.5	4.6	0.5
Inflex 2	0.55	0.08	0.45	0.08	0.57	0.09	0.57	0.09
Mean AMP (dB)	56.2	1.8	58.0	1.8	56.6	2.1	56.6	2.1
Max AMP (dB)	66.8	1.7	68.4	1.7	65.2	2.1	66.7	2.1
Int CV (dB)	30.7	1.0	29.0	1.0	31.3	1.4	27.8	1.4
**HNR (dB)**	**7.0**	**0.9**	**10.8**	**0.9**	10.8	1.0	11.9	1.0
**Jitter (Hz)**	**0.026**	**0.003**	**0.018**	**0.003**	0.022	0.003	0.020	0.003
Shimmer (dB)	0.159	0.010	0.137	0.010	0.114	0.008	0.125	0.008
Dur (s)	1.3	0.3	1.4	0.3	1.2	0.3	2.3	0.3
Dur Vocal (s)	1.0	0.2	1.1	0.2	1.1	0.2	1.4	0.2
% NLP	6.2	2.6	5.2	2.6	10.8	3.1	3.7	3.1

Linear mixed models (LMMs) and resulting estimated marginal means (EMMs ± standard error of the mean, *sem*; significant differences bolded) comparing voice parameters between deaf and normally hearing men and women in the fear context. All models, dependent variable: the given voice parameter; random variable (with intercept): vocalizer ID; fixed variables: deafness (impaired hearing or normal hearing). For all LMMs, model intercepts were significant at p<.001. Significant effects in LMMs were further examined using pairwise tests with Šidák correction for multiple comparisons. All tests two-tailed, alpha 0.05, where *** p<.001; ** p<.01; * p<.05; †p<.10. Data derive from n=120 vocalizers with an even sex ratio (60 deaf; 60 controls); for a small number of voice stimuli, specific acoustic parameters could not be measured, for instance, f_o parameters in the presence of severe deterministic chaos (see Methods).

Supplementary Table 10. Linear Mixed Models: Formant spacing (ΔF) comparisons between deaf and normally hearing men (Deafness) by emotional context (Context).

Source	*df1*, *df2*	*F*	*p*
**Final Model (a)**			
Intercept	1, 51.1	10718.9	<.001
Deafness	1, 51.1	4.3	.043
Context	2, 114.9	2.3	.108
Deafness * Context	2, 114.9	7.2	.001
**Final Models (b)**			
Intercept (aggression)	1, 34.5	5181.6	<.001
Deafness	1, 34.5	12.1	.003
Intercept (pain)	1, 38.3	5270.7	<.001
Deafness	1, 38.3	3.2	.084
Intercept (fear)	1, 31.8	4700.2	<.001
Deafness	1, 31.8	0.3	.561

All models, dependent variable: Formant spacing (ΔF) measured from the first four formants $F1$-$F4$ in men's vocalizations; random variable (with intercept): vocalizer ID. Final models a, fixed variables: Deafness * emotional context, N=140 calls with f_o < 400 Hz. Final models b, fixed variable: deafness (split by context; n=43 aggressive calls, 51 pain calls, 46 fear calls).

Supplementary Table 11. Linear Mixed Models: Apparent vocal tract length (VTL) comparisons between deaf and normally hearing men (Deafness) by emotional context (Context).

Source	*df1*, *df2*	*F*	*p*
**Final Model (a)**			
Intercept	1, 53	10001.6	<.001
Deafness	1, 53	4.7	.034
Context	2, 117	2.3	.107
Deafness * Context	2, 117	7.1	.001
**Final Models (b)**			
Intercept (aggression)	1, 33.9	4488.7	<.001
Deafness	1, 33.9	10.7	.003
Intercept (pain)	1, 39.7	4444.103	<.001
Deafness	1, 39.7	3.8	.058
Intercept (fear)	1, 31.3	4990.0	<.001
Deafness	1, 31.3	0.5	.491

All models, dependent variable: apparent vocal tract length (VTL) measured from the first four formants $F1$-$F4$ in men's vocalizations; random variable (with intercept): vocalizer ID. Final models a, fixed variables: Deafness * emotional context, N=140 calls where f_o < 400 Hz. Final models b, fixed variable: Deafness (split by context; n=43 aggressive calls, 51 pain calls, 46 fear calls).

Supplementary Table 12. Linear Mixed Models: Comparing positions of the first four formant frequencies (*F*1-*F*4) between deaf and normally hearing men (Deafness) by emotional context (Context).

Source	*df1, df2*	*F*	*p*
**Final Model (formant *F*1) (a)**		Male vocalizers	
Intercept	1, 37.7	1597.1	<.001
Deafness	1, 37.7	1.0	.319
Context	2, 114.1	0.7	.493
Deafness * context	2, 114.1	1.3	.264
**Final Model (formant *F*2) (a)**			
Intercept	1, 44.4	3575.5	<.001
Deafness	1, 44.4	25.0	<.001
Context	2, 115.3	1.1	.325
Deafness * context	2, 115.3	1.1	.326
**Final Models (formant *F*3) (a)**			
Intercept	1, 45.9	6488.9	<.001
Deafness	1, 45.9	3.1	.086
Context	2, 107.5	1.1	.345
Deafness * context	2, 107.5	6.0	.003
**Final Models (formant *F*3) (b)**			
Intercept (aggression)	1, 35.1	4266.5	<.001
Deafness	1, 35.1	8.7	.006
Intercept (pain)	1, 38.2	3484.3	<.001
Deafness	1, 38.2	2.7	.110
Intercept (fear)	1, 31.7	2808.6	<.001
Deafness	1, 31.7	0.5	.497
**Final Models (formant *F*4) (a)**			
Intercept	1, 49.1	8741.9	<.001
Deafness	1, 49.1	.662	.420
Context	2, 113.8	2.8	.062
Deafness * context	2, 113.8	6.4	.002
**Final Models (formant *F*4) (b)**			
Intercept (aggression)	1, 32.9	3702.9	<.001
Deafness	1, 32.9	6.1	.019
Intercept (pain)	1, 38.5	4002.5	<.001
Deafness	1, 38.5	1.1	.295
Intercept (fear)	1, 31.4	4994.2	<.001
Deafness	1, 31.4	2.1	.159

Dependent variables: First four formants (*F*1 to *F*4) measured from men's vocalizations; random model variable (with intercept): vocalizer ID. Final models a, fixed variables: Deafness * emotional context; *N*=140 calls where f_o < 400 Hz. Final models b, fixed variables: Deafness (split by context; *n*=43 aggressive calls, 51 pain calls, 46 fear calls).

Supplementary Table 13a. Linear Mixed Models (LMM): Forced-choice emotion classification (Experiment 1) comparing listeners' correct discriminations of intended emotion (aggression, fear, pain) for deaf versus normally hearing vocalizers (Deafness) in each emotional context (Context).

Source	*df1, df2*	*F*	*p*	*df1, df2*	*F*	*p*
**Final Models (a)**	Male vocalizers			Female vocalizers		
Intercept	1, 8334	9535.4	<.001	1, 8334	9326.0	<.001
Deafness	1, 8334	324.3	<.001	1, 8334	176.9	<.001
Context	2, 8334	64.1	<.001	2, 8334	32.5	<.001
Deafness * Context	2, 8334	228.2	<.001	2, 8334	87.3	<.001
**Final Models (b)**						
Intercept (aggression)	1, 2795.6	3111.9	<.001	1, 2808.4	2643.8	<.001
Deafness	1, 2795.6	944.9	<.001	1, 2808.4	348.8	<.001
Intercept (pain)	1, 2796.0	4008.5	<.001	1, 2768.1	3166.0	<.001
Deafness	1, 2796.0	3.5	.063	1, 2768.1	27.0	<.001
Intercept (fear)	1, 2769.8	2548.9	<.001	1, 2781.9	3538.9	<.001
Deafness	1, 2769.8	1.8	.177	1, 2781.9	0.1	.791
**Omnibus Model**	Both vocalizer sexes					
Intercept	1, 16656	18179.4	<.001			
Deafness	1, 16656	473.3	<.001			
Context	2, 16656	67.2	<.001			
Vocalizer sex	1, 16656	1.7	.196			
Listener Sex	1, 16656	1.9	.168			
Deafness * context	2, 16656	282.5	<.001			
Deafness * vocalizer sex	1, 16656	9.4	.002			
Deafness * listener sex	1, 16656	0.1	.784			
Context * vocalizer sex	2, 16656	27.3	<.001			
Context * listener sex	2, 16656	1.9	.146			
Speaker sex * listener sex	1, 16656	1.5	.225			
Deafness * context * vocalizer sex	2, 16656	19.5	<.001			
Deafness * context * listener sex	2, 16656	0.1	.948			
Deafness * vocalizer sex * listener sex	1, 16656	0.1	.766			
Context * vocalizer sex * listener sex	2, 16656	3.0	.052			
Deafness * context * vocalizer sex * listener sex	2, 16656	0.1	.912			

All models, dependent variable: Correct discrimination of intended emotion (proportion); random variables (with intercept): listener ID * vocalizer ID. Omnibus model, fixed variables: deafness * emotional context * vocalizer sex * listener sex. Final models a, fixed variables: deafness * emotional context (split by vocalizer sex); Final models b, fixed variable: deafness (split by emotional context and vocalizer sex). Experiment 1. *n*=139 listeners.

Supplementary Table 13b. Generalized Linear Mixed Models (GLMM), binary logistic regression. Forced-choice emotion classification (Experiment 1) comparing listeners' correct discriminations of intended emotion (aggression, fear, pain) for deaf versus normally hearing vocalizers (Deafness) in each emotional context (Context).

Source	*df1, df2*	*F*	*p*	*df1, df2*	*F*	*p*
**Final Models (a)**	Male vocalizers			Female vocalizers		
Intercept	5, 8334	146.4	<.001	5, 8334	74.9	<.001
Deafness	1, 8334	315.1	<.001	1, 8334	171.6	<.001
Context	2, 8334	61.2	<.001	2, 8334	31.9	<.001
Deafness * Context	2, 8334	206.9	<.001	2, 8334	84.4	<.001
**Final Models (b)**						
Intercept (aggression)	1, 2778	637.7	<.001	1, 2778	297.9	<.001
Deafness	1, 2778	637.7	<.001	1, 2778	297.9	<.001
Intercept (pain)	1, 2778	3.5	.061	1, 2778	26.8	<.001
Deafness	1, 2778	3.5	.061	1, 2778	26.8	<.001
Intercept (fear)	1, 2778	1.9	.171	1, 2778	0.1	.788
Deafness	1, 2778	1.9	.171	1, 2778	0.1	.788
**Omnibus Model**	Both vocalizer sexes					
Intercept	23, 16656	48.5	<.001			
Deafness	1, 16656	466.1	<.001			
Context	2, 16656	67.4	<.001			
Vocalizer sex	1, 16656	2.5	.113			
Listener Sex	1, 16656	1.6	.210			
Deafness * context	2, 16656	268.9	<.001			
Deafness * vocalizer sex	1, 16656	13.3	<.001			
Deafness * listener sex	1, 16656	0.2	.663			
Context * vocalizer sex	2, 16656	25.0	<.001			
Context * listener sex	2, 16656	2.3	.102			
Speaker sex * listener sex	1, 16656	1.4	.238			
Deafness * context * vocalizer sex	2, 16656	22.0	<.001			
Deafness * context * listener sex	2, 16656	0.1	.897			
Deafness * vocalizer sex * listener sex	1, 16656	0.1	.791			
Context * vocalizer sex * listener sex	2, 16656	2.5	.083			
Deafness * context * vocalizer sex * listener sex	2, 16656	0.1	.926			

All models, dependent variable: Correct discrimination of intended emotion (binary coding: 0-incorrect, 1-correct); random effects (with intercept): listener ID. Omnibus model, fixed effects: deafness * emotional context * vocalizer sex * listener sex. Final models a, fixed variables: deafness * emotional context (split by vocalizer sex); Final models b, fixed variable: deafness (split by emotional context and vocalizer sex). Experiment 1. *n*=139 listeners.

Supplementary Table 14. Mean raw hit rates and unbiased hit rates (Hu scores, untransformed and arcsine transformed) computed for the forced-choice emotion classification task (Experiment 1) comparing listeners' correct discriminations of intended emotion (aggression, fear, pain) for deaf versus normally hearing vocalizers in each emotional context.

Male vocalizers

Context	Group	Raw hit rate		Unbiased hit rate (Hu)		Unbiased hit rate (Hu, arcsine transformed)	
		Mean	*SEM*	*Mean*	*SEM*	*Mean*	*SEM*
Aggression	Control	0.71	0.012	0.42	0.01	0.70	0.01
	Deaf	0.20	0.012	0.11	0.01	0.30	0.02
Fear	Control	0.49	0.013	0.33	0.01	0.60	0.02
	Deaf	0.47	0.013	0.19	0.01	0.44	0.01
Pain	Control	0.61	0.013	0.41	0.01	0.68	0.02
	Deaf	0.57	0.013	0.26	0.01	0.52	0.01

Female vocalizers

Aggression	Control	0.62	0.013	0.42	0.01	0.70	0.01
	Deaf	0.29	0.013	0.18	0.01	0.41	0.01
Fear	Control	0.56	0.013	0.34	0.01	0.61	0.01
	Deaf	0.56	0.013	0.26	0.01	0.52	0.01
Pain	Control	0.58	0.013	0.34	0.01	0.61	0.01
	Deaf	0.48	0.013	0.21	0.01	0.47	0.01

Supplementary Table 15. Wilcoxon signed-rank tests on arcsine-transformed Hu scores comparing accuracy in listeners' discriminations of intended emotion (aggression, fear, pain) for deaf versus normally hearing vocalizers in each emotional context.

Context	Male vocalizers		Female vocalizers	
	Z	*p*	Z	*p*
Aggression	9.7	<.001	9.44	<.001
Fear	7.3	<.001	5.8	<.001
Pain	7.5	<.001	7.2	<.001

Supplementary Table 16. Linear Mixed Models: Authenticity identification (Experiment 3) comparing listeners' perceptions of authenticity for each intended emotion (aggression, fear, pain) for deaf versus normally hearing vocalizers (Deafness) and by emotional context (Context).

Source	*df1*, *df2*	*F*	*p*	*df1*. *df2*	*F*	*p*
**Final Models (a)**		Male vocalizers			Female vocalizers	
Intercept	1, 2338	22091.3	<.001	1, 2338	21701.5	<.001
Deafness	1, 2338	565.2	<.001	1. 2338	120.6	<.001
Context	2, 4676	105.9	<.001	2. 4676	210.6	<.001
Deafness * context	2, 4676	155.0	<.001	2. 4676	96.6	<.001
**Final Models (b)**						
Intercept (aggression)	1, 2338	9095.3	<.001	1, 2338	7064.2	<.001
Deafness	1, 2338	804.3	<.001	1, 2338	190.7	<.001
Intercept (pain)	1, 2338	12726.4	<.001	1, 2338	13807.8	<.001
Deafness	1, 2338	196.1	<.001	1, 2338	122.5	<.001
Intercept (fear)	1, 2338	8763.6	<.001	1, 2338	12154.5	<.001
Deafness	1, 2338	36.5	<.001	1, 2338	3.8	.052
**Omnibus Model**		Both vocalizer sexes				
Intercept	1, 4672	43679.1	<.001			
Deafness	1, 4672	596.5	<.001			
Context	2, 9344	236.5	<.001			
Vocalizer sex	1, 4672	12.6	<.001			
Listener Sex	1, 4672	2.3	.132			
Deafness * context	2, 9344	234.2	<.001			
Deafness * vocalizer sex	1, 4672	73.2	<.001			
Deafness * listener sex	1. 4672	2.3	.130			
Context * vocalizer sex	2, 9344	75.5	<.001			
Context * listener sex	2, 9344	2.7	.067			
Speaker sex * listener sex	1, 4672	0.5	.475			
Deafness * context * vocalizer sex	2, 9344	16.8	<.001			
Deafness * context * listener sex	2, 9344	1.9	.156			
Deafness * vocalizer sex * listener sex	1, 4672	1.4	.241			
Context * vocalizer sex * listener sex	2, 9344	0.2	.789			
Deafness * context * vocalizer sex * listener sex	2, 9344	0.5	.619			

All models, dependent variable: Authenticity rating (1 not all – 7 completely); random variables (with intercept): listener ID * vocalizer ID. Omnibus model, fixed variables: deafness * emotional context * vocalizer sex * listener sex. Final models a, fixed variables: deafness * emotional context (split by vocalizer sex); Final models b, fixed variable: deafness (split by context and vocalizer sex). Experiment 3. *n*=117 listeners.

Supplementary Table 17a. Linear Mixed Models: Deafness detection (Experiment 4) testing listeners' abilities to correctly identify the hearing deficits of deaf vocalizers for each emotional context (Context).

Source	*df1*, *df2*	*F*	*p*	*df1*. *df2*	*F*	*p*
**Final Models (a)**	Male vocalizers			Female vocalizers		
Intercept	1, 2740.2	11957.0	<.001	1, 2739.0	10718.8	<.001
Context	2, 5477.9	24.5	<.001	2, 5478.0	6.7	.001
**Omnibus Model**	Both vocalizer sexes					
Intercept	1, 5476.7	21256.5	<.001			
Context	1, 10951.6	19.3	<.001			
Vocalizer sex	1, 5476.7	9.1	.003			
Listener Sex	1, 5476.7	1.1	.286			
Context * vocalizer sex	2, 10951.6	8.3	<.001			
Context * listener sex	2, 10951.6	0.9	.388			
Speaker sex * listener sex	1, 5476.7	0.1	.850			
Context * vocalizer sex * listener sex	2, 10951.6	0.2	.783			

All models, dependent variable: Correct detection of deafness status (proportion); random variables (with intercept): listener ID * vocalizer ID. Omnibus model, fixed variables: Emotional context * vocalizer sex * listener sex. Final model, fixed variable: Emotional context (split by vocalizer sex). Experiment 4. *n*=137 listeners.

Supplementary Table 17b. Generalized Linear Mixed Models (GLMM), binary logistic regression. Deafness detection (Experiment 4) testing listeners' abilities to correctly identify the hearing deficits of deaf vocalizers for each emotional context (Context).

Source	*df1*, *df2*	*F*	*p*	*df1*. *df2*	*F*	*p*
**Final Models (a)**	Male vocalizers			Female vocalizers		
Intercept	2, 8216	20.54	<.001	2, 8217	5.7	.003
Context	2, 8216	20.54	<.001	2, 8217	5.7	.003
**Omnibus Model**	Both vocalizer sexes					
Intercept	11, 16427	6.2	<.001			
Context	2, 16427	16.6	<.001			
Vocalizer sex	1, 16427	12.5	<.001			
Listener Sex	1, 16427	0.6	.449			
Context * vocalizer sex	2, 16427	7.2	<.001			
Context * listener sex	2, 16427	0.8	.439			
Speaker sex * listener sex	1, 16427	0.1	.822			
Context * vocalizer sex * listener sex	2, 16427	0.2	.824			

All models, dependent variable: Correct detection of deafness status (binary coding: 0-incorrect, 1-correct); random variables (with intercept): listener ID. Omnibus model, fixed variables: Emotional context * vocalizer sex * listener sex. Final model, fixed variable: Emotional context (split by vocalizer sex). Experiment 4. *n*=137 listeners.

Supplementary Table 18. Linear Mixed Models: Forced-choice emotion classification (Experiment 1) comparing listeners' correct classification (raw hit rates) of intended emotion (aggression, fear, pain) for vocalizations of deaf vocalizers as a function of the severity of their deafness, for each emotional context (Context).

Source	*df1*, *df2*	*F*	*p*
**Final Models (a)**	Both vocalizer sexes		
Intercept	1, 8334	5891.6	<.001
Severity of deafness	1, 8334	35.6	<.001
Context	2, 8334	289.9	<.001
Severity of deafness * context	2, 8334	14.3	<.001
**Final Models (b)**			
Intercept (aggression)	1, 2795.3	749.1	<.001
Severity of deafness	1, 2795.3	30.8	<.001
Intercept (pain)	1, 2780.1	2664.0	<.001
Severity of deafness	1, 2780.1	35.4	<.001
Intercept (fear)	1, 2783.1	2701.1	<.001
Severity of deafness	1, 2783.1	0.7	.409

All models, dependent variable: Correct classification of intended emotion (proportion, raw hit rates); random variables (with intercept): listener ID * vocalizer ID. Final models a, fixed variables: severity of deafness * emotional context; Final models b, fixed variable: severity of deafness (split by context). Experiment 1. *n*=139 listeners. Severity of deafness categorization: *n*=20 congenitally deaf vocalizers with no prior auditory experience; *n*=40 deaf vocalizers with some limited prior acoustic experience (i.e., in early ontogeny or by use of hearing aid or cochlear implant).

Supplementary Table 19. Linear Mixed Models: Authenticity identification (Experiment 3) comparing listeners' correct discrimination of intended emotion (aggression, fear, pain) for vocalizations of deaf vocalizers as a function of the severity of their deafness, for each emotional context (Context).

Source	*df1, df2*	*F*	*p*
**Final Models (a)**	Both vocalizer sexes		
Intercept	1, 2338	12176.0	<.001
Severity of deafness	1, 2338	5.03	0.025
Context	2, 4676	327.1	<.001
Severity of deafness * context	2, 4676	.682	.506
**Final Models (b)**			
Intercept (aggression)	1, 2338	4314.5	<.001
Severity of deafness	1, 2338	4.5	.034
Intercept (pain)	1, 2338	8980.1	<.001
Severity of deafness	1, 2338	3.3	.068
Intercept (fear)	1, 2338	8527.5	<.001
Severity of deafness	1, 2338	.472	.492

All models, dependent variable: Authenticity rating (1-7); random variables (with intercept): listener ID * vocalizer ID. Final models a, fixed variables: severity of deafness * emotional context; Final models b, fixed variable: severity of deafness (split by context). Experiment 3. n=117 listeners. Severity of deafness categorization: n=20 congenitally deaf vocalizers with no prior auditory experience; n=40 deaf vocalizers with some limited prior acoustic experience (i.e., in early ontogeny or by use of hearing aid or cochlear implant).

Supplementary Table 20. Linear Mixed Models: Deafness detection (Experiment 4) testing listeners' ability to correctly identify the hearing status of deaf vocalizers as a function of the severity of their deafness, for each emotional context (Context).

Source	*df1*, *df2*	*F*	*p*
**Final Models (a)**		Both vocalizer sexes	
Intercept	1, 2738	9593.6	<.001
Severity of deafness	1, 2738	17.0	<.001
Context	2, 5476	20.1	<.001
Severity of deafness * context	2, 5476	4.1	.016
**Final Models (b)**			
Intercept (aggression)	1, 2738	4022.7	<.001
Severity of deafness	1, 2738	8.3	.004
Intercept (pain)	1, 2738	5429.4	<.001
Severity of deafness	1, 2738	.944	.331
Intercept (fear)	1, 2738	4037.1	<.001
Severity of deafness	1 2738	20.3	<.001

All models, dependent variable: Correct detection of deafness status (proportion); random variables (with intercept): listener ID * vocalizer ID. Final models a. fixed variables: severity of deafness * emotional context; Final models b, fixed variable: severity of deafness (split by context). Experiment 4. *n*=137 listeners. Severity of deafness categorization: *n*=20 congenitally deaf vocalizers with no prior auditory experience; *n*=40 deaf vocalizers with some limited prior acoustic experience (i.e., in early ontogeny or by use of hearing aid or cochlear implant).
