# Supplementary material for: Humans need auditory experience to produce typical volitional nonverbal vocalizations
Source: Commun Psychol. 2024 Jul 18;2:65. doi: 10.1038/s44271-024-00104-6 (PMC11332021; doi:10.1038/s44271-024-00104-6)
Supplement: Supplementary file 3 — Description of additional supplementary files [file 44271_2024_104_MOESM3_ESM.pdf]

Description Of Additional Supplementary File

File Name: Supplementary File 1

Description: Raw datasets in spreadsheet format containing data from 120 vocalisers (demographic data, acoustic analyses of vocalisations, survey data) and data from nearly 400 listeners (responses from four perception experiments).

File Name: Supplementary File 2

Description: Source data used in the figures in the manuscript.
