# Supplementary material for: Humans need auditory experience to produce typical volitional nonverbal vocalizations
Source: Commun Psychol. 2024 Jul 18;2:65. doi: 10.1038/s44271-024-00104-6 (PMC11332021; doi:10.1038/s44271-024-00104-6)
Supplement: Supplementary file 6 — Reporting Summary [file 44271_2024_104_MOESM6_ESM.pdf]

nature portfolio

Corresponding author(s): Katarzyna Pisanski

Last updated by author(s): Apr 22, 2024

Reporting Summary

Nature Portfolio wishes to improve the reproducibility of the work that we publish. This form provides structure for consistency and transparency in reporting. For further information on Nature Portfolio policies, see our Editorial Policies and the Editorial Policy Checklist.

Statistics

For all statistical analyses, confirm that the following items are present in the figure legend, table legend, main text, or Methods section.

n/a	Confirmed	
☐	☒	The exact sample size (*n*) for each experimental group/condition, given as a discrete number and unit of measurement
☐	☒	A statement on whether measurements were taken from distinct samples or whether the same sample was measured repeatedly
☐	☒	The statistical test(s) used AND whether they are one- or two-sided<br>*Only common tests should be described solely by name; describe more complex techniques in the Methods section.*
☐	☒	A description of all covariates tested
☐	☒	A description of any assumptions or corrections, such as tests of normality and adjustment for multiple comparisons
☐	☒	A full description of the statistical parameters including central tendency (e.g. means) or other basic estimates (e.g. regression coefficient) AND variation (e.g. standard deviation) or associated estimates of uncertainty (e.g. confidence intervals)
☐	☒	For null hypothesis testing, the test statistic (e.g. F, t, r) with confidence intervals, effect sizes, degrees of freedom and P value noted<br>*Give P values as exact values whenever suitable.*
☒	☐	For Bayesian analysis, information on the choice of priors and Markov chain Monte Carlo settings
☒	☐	For hierarchical and complex designs, identification of the appropriate level for tests and full reporting of outcomes
☐	☒	Estimates of effect sizes (e.g. Cohen's d, Pearson's r), indicating how they were calculated

Our web collection on statistics for biologists contains articles on many of the points above.

Software and code

Policy information about availability of computer code

Data collection	Acoustic analyses were performed using open-source acoustic analysis software Praat v 6.1.21 and the R package soundgen. All perception and rating experiments were conducted using a custom computer interface designed using the heroku app (https://www.heroku.com/). Auditory screening tests were conducted in the lab using a web-based computer platform (e-audiologia.pl) (see Masalski & Kręcicki, 2013; Masalski et al., 2014). Word clouds (Supplementary Figure 2) were created using the website wordcloud.com.
Data analysis	Data analysis was performed using R v 4.3.2 and SPSS v 25

For manuscripts utilizing custom algorithms or software that are central to the research but not yet described in published literature, software must be made available to editors and reviewers. We strongly encourage code deposition in a community repository (e.g. GitHub). See the Nature Portfolio guidelines for submitting code & software for further information.

March 2021

Data

Policy information about availability of data

All manuscripts must include a data availability statement. This statement should provide the following information, where applicable:

- Accession codes, unique identifiers, or web links for publicly available datasets
- A description of any restrictions on data availability
- For clinical datasets or third party data, please ensure that the statement adheres to our policy

All anonymized data are included in this published article (and its supplementary information files) and on the Open Science Framework (https://doi.org/10.17605/OSF.IO/CJNME), including quantitative measures pertaining to acoustic analyses of vocalizations and coded listener responses from perception experiments.

Human research participants

Policy information about studies involving human research participants and Sex and Gender in Research.

Reporting on sex and gender	Data were collected with an aim to obtain balanced sex ratios, wherein sex was based on self-report. Vocalizers thus included 60 men (30 hearing-impaired, 30 typically hearing) and 60 women (30 hearing-impaired, 30 typically hearing). In perception experiments involving 444 participants, 59% of raters self-reported as women (Total 264 women, 180 men). The exact break-down by perception experiment was as follows: Forced-choice emotion classification (82 women, 57 men); Open-ended emotion identification (36 women, 15 men); Authenticity identification (61 women, 56 men); Deafness detection (85 women, 52 men). Where relevant (i.e. where results differ between men and women), analyses are reported separately for each sex.
Population characteristics	The sample of vocalizers included 60 profoundly deaf adults (30 men, mean age ± sd 29±11.7, age range 16-53 years; 30 women, mean age 30±11.6, age range 17-52 years) and 60 typically hearing controls matched by sex, age, and education level to the deaf sample (30 men, age 30±10.6, range 16-55 years; 30 women, age 30±11.2, range 19-55 years). Table 1 provides detailed sample descriptives including onset, duration and causes of deafness. Raters taking part in perception experiments reported normal hearing and were between 16 and 60 years old (see Supplementary Table 3 for sample descriptives of adult listeners taking part in four independent perception experiments).
Recruitment	Deaf participants were recruited via advertisements and professional contacts with local associations or specialized schools for deaf persons. Age-sex-matched hearing controls were recruited from the general population and local community through personal and professional contacts. To take part in the study, controls must have declared normal hearing whereas deaf participants must have received a clinical diagnosis of bilateral profound deafness, which we confirmed prior to their taking part in the study.<br>Raters in perception experiments were recruited from the general population and local community via online advertisements, posters and professional contacts.<br>All participants were recruited from industrialized countries, and thus our results may be specific to "WEIRD" populations and should be replicated on a broader representation of human cultures including small-scale and marginalized societies.
Ethics oversight	This research was approved by the Institutional Review Board at the University of Wrocław (IPE0021) in consultation with the Polish Association of the Deaf. The research was performed in accordance with the Declaration of Helsinki on Biomedical Studies Involving Human Subjects. Informed and written consent were obtained from all participants.

Note that full information on the approval of the study protocol must also be provided in the manuscript.

Field-specific reporting

Please select the one below that is the best fit for your research. If you are not sure, read the appropriate sections before making your selection.

☐ Life sciences ☒ Behavioural & social sciences ☐ Ecological, evolutionary & environmental sciences

For a reference copy of the document with all sections, see nature.com/documents/nr-reporting-summary-flat.pdf

Behavioural & social sciences study design

All studies must disclose on these points even when the disclosure is negative.

Study description	Data are quantitative
Research sample	The sample of vocalizers included adults of a broad age range and socioeconomic background, half of whom were hearing-impaired, and were recruited from the local community near Wroclaw, Poland. Raters in perception experiment were also adults of a broad age range with typical hearing and were recruited from the general population and local community. The sample of raters included a

minority of students. Additional details regarding sample sizes, sex, age and recruitment methods are given above (see Human Research Participants).

Sampling strategy	Sampling of vocalizers was random within the special population (i.e. hearing-impaired participants) and pseudo-random for sex-age-matched hearing controls, as controls were matched by sex and age to our sample of hearing-impaired adults. Sampling of raters was pseudo-random as professional contacts were one of the methods used to recruit raters.<br>For perception experiments, sample sizes were pre-determined prior to experimentation: for forced-choice perception experiments (experiments 1,3,4), we created a stopping rule for data collection at approximately 30 raters (15 male and 15 female) per voice stimulus, based on evidence that 15 raters per sex produces Cronbach's alphas exceeding 0.8 indicating a high degree of inter-rater agreement and consistency in ratings. Due to random sampling for playback, some voice stimuli reached (and thus exceeded) 30 ratings earlier than did others; data were collected until minima were reached for all voice stimuli. For perception experiment 2 in which participants provided open-ended responses, the stopping rule was set to 50 listeners for a representative sample.
Data collection	All vocalizers (n=120) were recorded privately in a quiet room using a Tascam DR05 recorder at a sampling rate of 48kHz and 24-bit amplitude quantification, positioned at a distance of 150 cm to avoid audio clipping of high-amplitude vocalizations. Microphone distance and input levels were standardized between and within vocalizers. Instructions were given to all participants in written form prior to voice recording, and for deaf participants were also provided in sign language via a pre-recorded video featuring a Professional Sign Language Interpreter, who was also available in person on site if additional communication or translation was required. Participants were instructed to imagine themselves in three scripted contexts, presented in a random order (aggression, fear, and pain) and to respond vocally and nonverbally (without the use of words) to each given scenario, producing one vocal output per scenario. The researcher explained the task, but left the room during the audio recordings, to ensure that vocalizers felt maximally comfortable producing the vocalizations.<br>In lab-based perception experiments, raters performed the study through a custom interface and professional headphones. The researcher was present but did not interfere unless the rater had questions.<br>Researchers were blind to the experiment (which was randomly selected for each rater) and conditions in all perception experiments. Researchers were also blind to the hearing status of the vocalizer and the context condition when performing acoustic analyses of vocalizations.
Timing	Voice recordings were collected between October 2018 and February 2019. Playback experiments were conducted between October 2019 and February 2020.
Data exclusions	A sample of vocal stimuli were selected for formant analysis. Otherwise, there were no other data exclusions.<br>Because formants require a relatively dense harmonic structure to be measured reliably, we selected vocalizations with a relatively low mean fo (< 400 Hz). Moreover, because too few women produced such relatively low-pitched vocalizations, our analyses focused on males.
Non-participation	No participants dropped out or declined participation.
Randomization	Allocation of raters to a given perception experiment (1 through 4) was random (as drawn by a computer algorithm).

Reporting for specific materials, systems and methods

We require information from authors about some types of materials, experimental systems and methods used in many studies. Here, indicate whether each material, system or method listed is relevant to your study. If you are not sure if a list item applies to your research, read the appropriate section before selecting a response.

Materials & experimental systems

n/a	Involved in the study	
☒	☐	Antibodies
☒	☐	Eukaryotic cell lines
☒	☐	Palaeontology and archaeology
☒	☐	Animals and other organisms
☒	☐	Clinical data
☒	☐	Dual use research of concern

Methods

n/a	Involved in the study	
☒	☐	ChIP-seq
☒	☐	Flow cytometry
☒	☐	MRI-based neuroimaging
